# Severe Hyperprolactinemia Promotes Brown Adipose Tissue Whitening and Aggravates High Fat Diet Induced Metabolic Imbalance

**DOI:** 10.3389/fendo.2022.883092

**Published:** 2022-06-10

**Authors:** Felicitas Lopez-Vicchi, Catalina De Winne, Ana Maria Ornstein, Eleonora Sorianello, Judith Toneatto, Damasia Becu-Villalobos

**Affiliations:** Instituto de Biología y Medicina Experimental, Consejo Nacional de Investigaciones Científicas y Técnicas, Buenos Aires, Argentina

**Keywords:** prolactin, brown adipose tissue, thermogenic markers, whitening, UCP1, PGC1 alpha, CIDEA, obesity

## Abstract

**Background:**

The association of high serum prolactin and increased body weight is positive but controversial, therefore we hypothesized that additional factors such as diets and the impact of prolactin on brown adipose tissue may condition its metabolic effects.

**Methods:**

We used LacDrd2KO females with lifelong severe hyperprolactinemia due dopamine-D2 receptor deletion from lactotropes, and slow onset of metabolic disturbances, and compared them to their respective controls *(Drd2 ^loxP/loxP^*). Food intake, and binge eating was evaluated. We then challenged mice with a High Fat (HFD) or a Control Diet (CD) for 8 weeks, beginning at 3 months of age, when no differences in body weight are found between genotypes. At the end of the protocol brown and white adipose tissues were weighed, and thermogenic and lipogenic markers studied, using real time PCR (*Ucp1, Cidea, Pgc1a, Lpl, adiponectin, Prlr*) or immunohistochemistry (UCP1). Histochemical analysis of brown adipose tissue, and glucose tolerance tests were performed.

**Results:**

Hyperprolactinemic mice had increased food intake and binge eating behavior. Metabolic effects induced by a HFD were exacerbated in lacDrd2KO mice. Hyperprolactinemia aggravated HFD-induced body weight gain and glucose intolerance. In brown adipose tissue pronounced cellular *whitening* as well as decreased expression of the thermogenic markers *Ucp1* and *Pgc1a* were observed in response to high prolactin levels, regardless of the diet, and furthermore, hyperprolactinemia potentiated the decrease in *Cidea* mRNA expression induced by HFD. In subcutaneous white adipose tissue hyperprolactinemia synergistically increased tissue weight, while decreasing *Prlr*, Adiponectin and *Lpl* mRNA levels regardless of the diet.

**Conclusions:**

Pathological hyperprolactinemia has a strong impact in brown adipose tissue, lowering thermogenic markers and evoking tissue whitening. Furthermore, it modifies lipogenic markers in subcutaneous white adipose, and aggravates HFD-induced glucose intolerance and *Cidea* decrease. Therefore, severe high prolactin levels may target BAT function, and furthermore represent an adjuvant player in the development of obesity induced by high fat diets.

## Introduction

Prolactin is named after its major role in lactation in mammals, even though it participates in multiple biological processes including reproduction, osmoregulation, immunoregulation, growth and energy metabolism (1–3), consistent with the wide distribution of prolactin receptors (PRLR) in the body (4). *Prlr* mRNA is expressed in numerous tissues involved in the control of energy balance, such as adipose tissue, pancreas, small intestine, liver and brain, where prolactin integrates endogenous or environmental signals to ensure metabolic homeostasis through modulation of food intake, lipid and glucose metabolism (3).

These metabolic actions of prolactin are fundamental during pregnancy and lactation, however, the association between elevated prolactin levels and increased body weight, food intake or adiposity in other situations such as prolactinoma occurrence or psychiatric treatments that target the dopamine type 2 receptors (DRD2), remains controversial. Studies in rats show that chronic hyperprolactinemia induced by regular injections of dopamine antagonists or exogenous prolactin, or ectopic pituitary transplants, is associated with increased food intake and body weight (5–7), whereas pharmacological suppression of prolactin secretion with the dopamine-agonist bromocriptine evokes the opposite outcome, being most effective in lactating rats and least in males (8). On the other hand, male mice with ectopic pituitaries show only a small increase in body weight and food intake, and a slight decline in fat mass (8). In the same line, hyperprolactinemic dopamine receptor D2 knockout (*Drd2^-/-^*) female mice have similar body weight to controls (9, 10), whereas body weight is remarkably increased in after 6 months of age in hyperprolactinemic mice with selective deletion of the dopamine receptor D2 in lactotropes (lacDrd2KO mice) (9).

Clinical data show an association of high very prolactin levels with increased prevalence of obesity and metabolic disorders such as dyslipidemia, glucose intolerance, and insulin resistance, and many of these disorders are improved by normalizing prolactin levels with dopamine agonists (8, 11–17). Moreover, genetic variants of the *PRL* locus (6p22.2 – p21.3) have been associated with alterations in body mass index in genome-wide association studies (18), and SNPs located near the *PRLR* gene correlate with an increased risk for gestational diabetes and obesity (19) strengthening the connection between prolactin and metabolic disorders. However, there is no strong evidence to indicate that high circulating concentrations of prolactin are a major factor in human obesity, and furthermore, prolactin levels below physiological range in humans and rodents associate with metabolic diseases implying also a protective effect of normal prolactin levels in metabolism (3, 20).

Therefore, the association of pathologically high prolactin levels and increased body weight is controversial but positive, suggesting that prolactin favors weight gain but additional factors may enhance or condition its effects.

High-Fat Diets (HFD) are well-known obesogenic stimuli, and in some circumstances, other challenges may potentiate or aggravate their metabolic effects, highlighting the multifactorial causation of obesity. In this context, we wished to determine the simultaneous effects of pathologically chronic hyperprolactinemia and HFD on food intake, adipose tissue accretion and expression of thermogenic and lipogenic markers, as well as glucose homeostasis in female mice.

PRLR signaling exerts crucial roles in the development and function of two major players of body energy balance: adipose tissue and the endocrine pancreas. Even though in transgenic mice lacking prolactin no profound metabolic phenotype was demonstrated (21), mice with *Prlr* deficiency showed a worsening in the induction of obesity or streptozotocin evoked diabetes (20, 22). Ample evidence has shown that this hormone participates in lipid metabolism, adipogenesis and adipocyte differentiation, as well as in pancreas development, beta islet proliferation, insulin synthesis and release (23), and the net effect of prolactin depends on its serum levels (3).

Adipose tissue is a metabolically dynamic organ, and is categorized in energy storing white adipose tissue (WAT), thermogenic brown adipose tissue (BAT) and thermogenic brown-like adipocytes named brite/beige adipocytes that are scattered within WAT (24). WAT, the main reservoir of energy is mainly categorized as subcutaneous (scWAT) and visceral (VAT) adipose tissue. *Prlr* mRNA has been documented in white adipocytes (25, 26), and hyperprolactinemic mouse models have increased adiposity as a result of either decreased lipolysis (9, 27) or increased lipogenesis in WAT (5). Nevertheless, opposite roles have also been described for prolactin or PRLR on adipogenesis regulation (20, 25, 28, 29).

On the other hand, BAT is a highly vascularized organ composed of adipocytes that contain small lipid droplets and a large number of mitochondria capable of oxidizing chemical energy to produce heat, providing thermogenic capacity to this tissue which depends mainly on the presence of the proton transporter uncoupling protein 1 (UCP1) (30). The role of prolactin in BAT physiology has not been extensively studied. *Prlr* knockout mice have smaller BAT depots and pre-adipocytes derived from this tissue are not capable of differentiating into mature adipocytes, a phenotype which can be reversed by the ectopic expression of *Prlr* (31).

Metabolic plasticity in response to hormonal stimuli or environmental cues has been identified as a hallmark feature of adipose tissue (32). Besides the well known plastic process of beiging, diet-induced obesity in mice evokes vascular remodeling and functional hypoxia leading to a “whitening” phenotype in BAT, characterized by mitochondrial dysfunction and loss, lipid droplet accumulation, and decreased expression of *Ucp1* and Vascular endothelial growth factor, and tissue inflammation (33–36). BAT whitening is therefore associated to decreased thermogenic capacity and impaired energy balance, and the role of prolactin in this process has not been studied.

Finally, a reasonable body of evidence shows that prolactin signaling is implicated in the regulation of glucose homeostatic adaptations to pregnancy mainly through its impact in pancreatic islet cell physiology and glucose metabolism (37). In this sense, adequate levels of prolactin are beneficial for maintaining glucose homeostasis. However, pathologically high levels of prolactin may alter glucose tolerance, disrupt glucose signaling in pancreas, and induce insulin resistance (3).

We have recently described that lifelong hyperprolactinemia in lacDrd2KO female mice induced gradual metabolic alterations. Intriguingly, even though the elevated serum prolactin levels were observed since the first month of age, obesity was of late onset, beginning mildly in 6-month-old female mice and culminating in morbid obesity from 10 months of age onward (27, 38). The slow onset of the metabolic phenotype, led us to investigate whether exposure to a HFD might accelerate and intensify the late metabolic changes found in the hyperprolactinemic mouse model with special focus food intake, glucose homeostasis and adipose gene expression profiles, and the processes of whitening and beiging described in BAT and WAT, respectively.

Our results not only highlight the role of prolactin as a metabolic hormone, but also demonstrate that very high prolactin levels or exposure to a HFD in isolation may cause slight metabolic imbalance. However, the combination of these two challenges, or the combination of severe hyperprolactinemia with other metabolic challenges such as stress, anxiety, nutritional imbalance or endocrine disruptors, may lead to overweight or obesity.

## Materials and Methods

### Animals

Mice lacking expression of D2Rs in pituitary lactotropes (lacDrd2KO) were generated by crossing *Drd2^loxP/loxP^* mice (39) with transgenic mice expressing Cre recombinase driven by the mouse prolactin promoter, Tg(Prl-cre)^1Mrub^ (40) for ten generations. Tissue specificity of Cre expression in Tg(Prl-cre)^1Mrub^ transgenic mice was analyzed by real time PCR and *Cre* mRNA levels were highly expressed in the pituitary and very low or almost absent in the hypothalamus, liver, kidney, ovary and lung (9).

Breeding pairs of female *Drd2^loxP/loxP^* and male *Drd2^loxP/loxP^*.Tg(Prl-cre) mice were used to generate *Drd2^loxP/loxP^* (control) and *Drd2^loxP/loxP^*.Tg(Prl-cre) (lacDrd2KO) littermates, which were included in each experiment. LacDrd2KO and their *Drd2^loxP/loxP^* control littermates were congenic to C57BL/6J (n= 10). Mice of mixed genotypes were housed in groups of 4 or 5 in a temperature-controlled room (22-24°C) with lights on at 0700 h and off at 1900 h, and had free access to tap water and laboratory chow, except when indicated. Prolactin levels for *Drd2^loxP/loxP^* and LacDrd2KO female mice are significantly higher from the first month of life (9). Because in male mice there was a marginal increase in prolactin levels, and no differences in body or pituitary weight, fat mass depots or food intake (9), we used female mice in our experiments.

Mice were euthanized by decapitation at 5-6 months of age. Sera were collected for prolactin measurements. White (gonadal, mesenteric and inguinal subcutaneous) and brown (interscapular) adipose tissue (WAT and BAT, respectively) were excised and weighed and frozen in dry ice to be stored at - 80°C. Collected tissue was later processed for qPCR or immunohistochemistry as detailed below.

All experimental procedures were carried according to guidelines of the institutional animal care and use committee of the Institute of Biology and Experimental Medicine, Buenos Aires (in accordance with the Animal Welfare Assurance for the Institute of Biology and Experimental Medicine, Office of Laboratory Animal Welfare, NIH, A#5072-01) and were approved by the ethics committee of the Institute of Biology and Experimental Medicine (N°CE031/2019).

### Food Intake

Food intake was determined in individually caged five-month-old female mice of both genotypes (Drd2 *^loxP/loxP^* and lacDrd2KO). Mice were provided *ad libitum* with a known amount of regular chow pellets (5% fat, 19% protein, and 5% fiber by weight; 2.4 kcal/g). During 5 days, residual food was weighed daily at the same hour to calculate daily food intake (1500 h).

### Binge-Eating Protocol

A binge-eating model was used to analyze the integrity of hedonic brain circuits controlling consumption of energy-rich palatable food. Protocol was adapted from (41) and shows that robust binge-eating episodes occur when satiated mice have time-limited access to a palatable stimulus. There is a significant gradual escalating profile of consumption of a HFD pellet for 4 successive days which validates the binge-eating model. Briefly, 5-month-old lacDrd2KO and *Drd2^loxP/loxP^* mice were single-housed in clean cages three days before the experiment and then assigned into two experimental groups: i) Control diet (CD) *ad libitum* group, which received unlimited access to CD and was daily exposed to a pellet of CD in the home cage floor from 0900 to 1100 h; and ii) daily HFD access group, which received unlimited access to CD during the whole experiment and was daily exposed to a pellet of HFD in the home cage floor from 0900 h to 1100 h (N=6,6, for *Drd2 ^loxP/loxP^* and 6,5 for lacDrd2KO mice, fed a CD and a HFD, respectively). A preliminary group with free access to HFD and daily exposure to a pellet of HFD in the home cage floor from 0900 to 1100 h was also included to validate the protocol (HFD *ad libitum* group), and no episode of binge-eating was evidenced in this group (data not shown). Both CD (D12450H, 10 kcal% fat D12451 Match 17% Sucrose) and HFD (45 kcal% fat, cat. no: D124521) were provided by Research Diets Inc., USA. Food intake from the lid was recorded in the 2-h food access period during the 4 consecutive days, and was very low (between 0 and 0.05 g with no genotype differences (data not shown).

### HFD Protocol: Body Weight and Food Intake

Three-month-old mice of both genotypes were acclimatized for 7 days in individual cages and then assigned into the CD or HFD group according to similar body weight criteria within each genotype. Thereafter mice were fed *ad libitum* with HFD for 8 weeks (45 kcal% fat, cat. no: D12451 procured from Research Diets Inc., USA), or matching CD from Research diets Inc. (D12450H, 10 kcal% fat D12451 Match 17% Sucrose). Body weight and food intake were measured weekly. Fat, and caloric intake were calculated taking into consideration grams of ingested food and the percentage of fat and calories of each diet (24 g and 4.3 g fat % for HFD and CD respectively, and 4.76 and 3.85 kcal/g for HFD and CD, respectively).

### Glucose Tolerance Test

Glucose tolerance test (GTT) was performed in conscious lacDrd2KO and *Drd2^loxP/loxP^* mice two weeks before the end of the HFD protocol (mice were approximately 5 months of age). Briefly, after overnight fast (8 h), ip glucose solution (2 mg/g body weight) was administered. Blood glucose levels (2 μl of blood obtained from the tail of each mouse) were measured at 0, 15, 30, 60, and 120 min after glucose injection with a hand-held glucose monitor (Dex-II; Bayer).

### RNA Extraction and cDNA Synthesis From Adipose Tissue Depots

Gene expression was measured in adipose tissue from 5-month-old mice of both genotypes fed during the two previous months with HFD or CD. WAT and BAT from *Drd2 ^loxP/loxP^* and lacDrd2KO were processed for recovery of total RNA using TRIzol reagent (Invitrogen, Buenos Aires, Argentina). The RNA concentration was determined on the basis of absorbance at 260 nm. RNA (1µg) was reversed transcribed in 20 µl volume in the presence of 0.5 mM deoxy-NTPs, 0.25 µg/ul oligo(dT)15 primer (Biodynamics, Buenos Aires, Argentina), using MMLV reverse transcriptase kit (1 U, Promega Co, Madison, USA). The reverse transcriptase was omitted in control reactions; the absence of PCR-amplified DNA fragments in these samples indicated the isolation of RNA free of genomic DNA. cDNA was stored at - 20°C until used for qPCR.

### Real time PCR

Measurements were performed as previously described in (10). Sense and antisense oligonucleotide primers were designed by the use of PrimerBlast (http://www.ncbi.nlm.nih.gov/tools/primer-blast/). Oligonucleotides were obtained from Thermo Fisher, Buenos Aires, and the sequences are described in **Table 1**. Briefly, the reactions were performed by kinetic PCR using 5x HOT FIREPol^®^ EvaGreen^®^ qPCR Mix Plus (ROX) Solis Biodyne (9.4 µl containing HOT FIREPol^®^ DNA Polymerase, 5x EvaGreen qPCR buffer, **12.5 mM dNTPs,** EvaGreen^®^dye, ROX dye), 100 ng cDNA and 0.24 µM or 0.48 μM primers in a final volume of 10.4 µl. After denaturation at 95°C for 15 minutes, the cDNA products were amplified with 40 cycles. Cycle conditions were the following: denaturation 95°C for 30 s, annealing 63°C for scWAT and 58°C for BAT for 30 s, and extension 72°C for 30 s and optical reading stage was performed at 80°C for 33 s. The accumulating DNA products were monitored by the Bio-Rad sequence detection system (Bio-Rad Laboratories, Hercules, California, USA) and data were stored continuously during the reaction. The results were validated on the basis of the quality of dissociation curves (10) generated at the end of the PCR runs by ramping the temperature of the samples from 60 to 95°C, while continuously collecting fluorescence data. Product purity was confirmed by agarose gel electrophoresis. Each sample was analyzed in duplicate. Relative gene expression levels were calculated according to the comparative cycle threshold (CT) method. Results were analyzed relative to a housekeeping gene (cyclophilin) within the same sample to normalize for possible variations in starting RNA quality and quantity, and RT efficiency. Normalized target gene expression relative to cyclophilin was obtained by calculating the difference in CT values, the relative change in target transcripts being computed as 2^-ΔCT^. To validate the comparative CT method of relative quantification, the efficiencies of each target and housekeeping gene amplification (endogenous cyclophilin) were measured and shown to be approximately equal. Cyclophilin mRNA levels were analyzed independently and did not vary in any of the experimental groups.

**Table 1:** Sequence of primers used.

Gene	*Primers*	Sequence (5' - 3')
*Adiponectin*	Sense	ATCCTGGCCACAATGGCACA
* *	Antisense	CAAGAAGACCTGCATCTCCT
*Cyclophilin*	Sense	TTCTTCATAACCACAGTCAAGACC
* *	Antisense	ACCTTCCGTACCACATCCAT
*Cidea*	Sense	AACCATGACCGAAGTAGCCG
* *	Antisense	CCAGGCCAGTTGTGATGACT
*Il1b*	Sense	AACCTGCTGGTGTGTGACGTTC
	Antisense	CAGCACGAGGTCTTTTTGTTGT
*Lpl*	Sense	CCCTACAAAGTGTTCCATTACCAA
* *	Antisense	TTGTGTTGCTTGCCATCCTCA
*Pgc1a*	Sense	TCTCAGTAAGGGGCTGGTTG
* *	Antisense	TTCCGATTGGTCGCTACACC
*Prlr*	Sense	CACAGTAAATGCCACGAACG
* *	Antisense	GGCAACCATTTTACCCACAG
*Ucp1*	Sense	AGTACCCAAGCGTACCAAGC
* *	Antisense	GACCCGAGTCGCAGAAAAGA

### Morphometric Analysis

A comparative histological analysis of interscapular BAT depots from lacDrd2KO and *Drd2^loxP/loxP^* mice was performed to evaluate adipocyte architecture. A portion of adipose tissue depots was fixed in 10% formalin and embedded in paraffin. To determine adipocyte and lipid droplet size, histological sections (5 μm) were cut from paraffin-embedded tissue, mounted on microscope glass slides and dried overnight in an incubator at 37°C. General morphology was evaluated with hematoxylin and eosin (H&E) staining. Digital images were captured using a 40X magnification objective, a Zeiss Axiostar Plus microscope and a Canon Power Shot G6 digital camera. Adipocyte size was measured with ImageJ and counted per field at 40X magnification. Six fields were randomly selected from at least two different slides per mouse (n = 3 mice per experimental group), an average of 86 adipocytes per mouse were evaluated. Lipid droplets within adipocytes were counted and their size recorded (average droplets per adipocyte 15.5 + 9.4, range 1-75 droplets per adipocyte, total number of droplets evaluated 15992). Lipid droplets were classified according to their size as large (>8.01 μm^2^); medium (3.01 to 8 μm^2^); or small (<3 μm^2.^).

### Immunohistochemistry

Serial sections of interscapular BAT performed on the same tissue samples used for H&E staining were used for immunostaining of UCP1. Briefly, slides were first deparaffinized, and then an antigen retrieval protocol using heat was used to unmask the antigens (citrate buffer 0.01 M, pH 6.0). Endogenous peroxidase blocking and nonspecific tissue blocking were performed. Tissue sections were incubated with the primary antibody at 4°C in humidity chambers. UCP1 antibody (U6382, Sigma-Aldrich; RRID : AB_261838) was diluted in antibody diluent (Dako) at 1:500. A commercial kit to detect mouse and rabbit antibodies was used (DAKO LSAB + Kit, HRP, horse radish peroxidase, DAB, chromogen 3,3′-diaminobenzidine, Vector Laboratories, CA, USA). Slides were counterstained with hematoxylin (Biopur, Rosario, Argentina), dehydrated, cleared, and mounted. Serial cuts incubated in the absence of primary antibody were used as negative controls. Images were taken with a Nikon Eclipse E200 Microscope fitted with a Micrometric SE Premium (Nikon Corp., Japan) digital still camera at 40X magnifications. DAB staining quantification was performed in 8–10 fields of each preparation randomly selected (Number of preparations 3 per mouse (N= 3 mice per group), using ImageJ 1.42 software (NIH, Bethesda, MD, USA).

### Statistical Analysis

Results are expressed as means ± SEM. Differences between means were analyzed by the unpaired Student’s t-test (in the case of only two groups). Two-way ANOVA for independent measures (GraphPad Prism) was used to analyze tissue weight, and gene expression (for the effects of diet and genotype). Three Way ANOVA for repeated measures (Statistica) was used to analyze body weight (for the effects of genotype, diet and week) and GTT (for the effects of genotype, diet and time post glucose injection). If P of interaction was found to be significant, individual means were compared by Tukey’s honest significant difference test; if interaction was not significant, groups of means were analyzed by the same test. Parametric or non-parametric comparisons were used as dictated by data distribution. P≤0.05 was considered significant.

## Results

### Characterization of Eating Behavior in 5-Month-Old lacDrd2KO and *Drd2^loxP/loxP^* Mice

**Figure 1:**
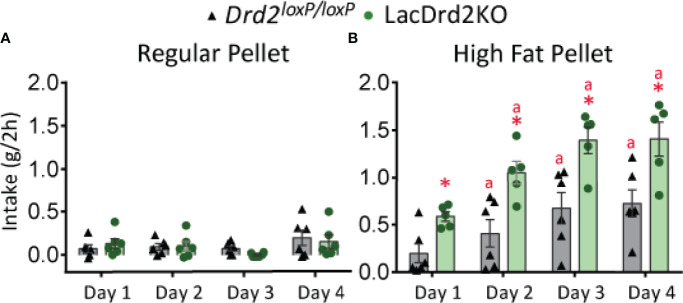
LacDrd2KO female mice have increased binge eating behavior. Characterization of binge-eating behavior in 5-month old lacDrd2KO and *Drd2^loxP/loxP^* female mice. **(A)** Regular pellet intake in grams during 2 hours for 4 consecutive days, n= 6 and 6 for *Drd2^loxP/loxP^* and lacDrd2KO mice, respectively. **(B)** High Fat pellet intake in grams during 2 hours for 4 consecutive days, n= 6 and 5 for Drd2loxP*^/loxP^* and lacDrd2KO mice, respectively. Repeated measures TWO ANOVA; “a” *P* ≤ 0.05 vs. genotype-matched HF mice on day 1; *P ≤ 0.05 vs. *Drd2^loxP/loxP^* HF- fed, day-matched mice.

Food intake is regulated by an integrated system involving both homeostatic and hedonic brain circuits (42). In accordance with previous data obtained in our laboratory (9), food intake was increased in lacDrd2KO compared to *Drd2 ^loxP/loxP^* female mice at 5 months of age, indicating alterations in homeostatic brain circuits regulating appetite (*Drd2 ^loxP/loxP^* = 3.2 + 0.2 and lacDrd2KO = 4.1 + 0.2 g/day, N 8,8, *P* ≤ 0.01). Moreover, a binge-eating model induced by daily and time-limited access to a pellet of HFD was used to analyze the integrity of hedonic brain circuits controlling consumption of energy-rich palatable food. Five-month-old satiated lacDrd2KO and *Drd2 ^loxP/loxP^* mice ate similar grams of CD pellet when daily exposed for 2-h, the difference g eaten was not significant between genotypes due to the short period analyzed during daytime (**Figure 1A**). On the other hand, satiated mice of both genotypes with 2-h daily access to a pellet of HFD displayed a significant gradual escalating profile of consumption for 4 successive days (**Figure 1B**, Interaction genotype x day, *P*=0.62, F_3,36 =_ 0.59; days 2 to 4 different from day 1 P< 0.005), validating that binge-eating episodes occur when there is time-limited access to a palatable stimulus. Most interestingly, lacDrd2KO female mice ate a significantly higher amount of fat-enriched pellet compared to diet-matched *Drd2 ^loxP/loxP^* control mice (main effect genotype, *P* < 0.0001, **Figure 1B**).

### Differential Effect of a High-Fat Diet on Body Weight and Food Intake in lacDrd2KO Compared to *Drd2 ^loxP/loxP^* Mice

**Figure 2:**
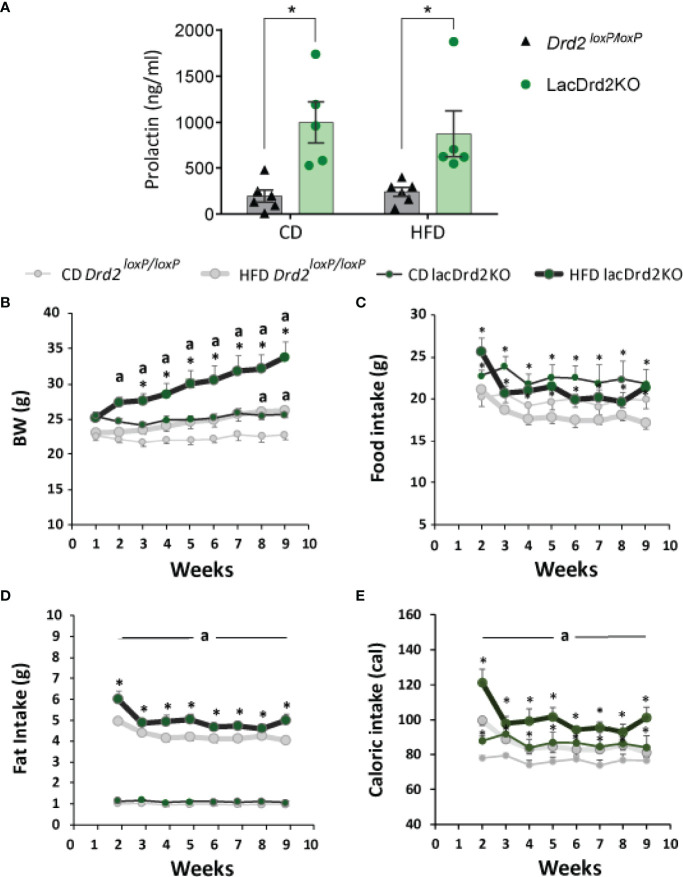
Serum prolactin, body weight and food intake in response to a high-fat diet and hyperprolactinemia. **(A)** Serum prolactin levels in ng/ml at the end to the feeding protocol (Two Way Anova, *P ≤0.05 vs. diet- matched *Drd2 ^loxP/loxP^* mice). **(B)** Weekly measured body weight in grams; **(C)** food intake in grams (g); **(D)** fat intake in grams (g); and **(E)** energy intake in calories consumed (cal) (n= 6, and 6 *Drd2 ^loxP/loxP^* control diet (CD) and high fat diet (HFD) respectively, and 5 and 5 for lacDrd2KO mice fed with CD or HFD, respectively. Three-way ANOVA, “a” = *P* ≤ 0. 05 vs. genotype- and time- matched mice fed with CD *P ≤0.05 vs diet- and time-matched *Drd2 ^loxP/loxP^* mice.

Animals were exposed to HFD or CD for 8 weeks. At the end of the experiment prolactin levels were significantly higher in LacDrd2KO compared to *Drd2^loxP/loxP^* female mice, as expected, and no effect of the diet was found (interaction genotype x diet F_1,18 =_ 0.30, *P*=0.59, NS; main effect of genotype, *P*=0.0002, **Figure 2A**), these values in lacDrd2KO mice are similar to those found in experimental prolactinomas or during lactation peaks.

Both *Drd2loxP/loxP* and lacDrd2KO mice fed a HFD showed a significant increase in body weight, but the increase was of a greater magnitude and evidenced earlier in lacDrd2KO mice (significant differences were detected from week 2 onwards in lacDrd2KO and from week 8 onwards in Drd*2^loxP/loxP^* mice, compared to genotype-matched CD mice, **Figure 2B**). Furthermore, body weight was significantly higher in HFD lackDrd2KO mice compared to HFD *Drd2^loxP/loxP^* mice from week 3 onwards (*P*< 0.02 from week 3 to week 9), and the difference in body weight gain induced by HFD between genotypes increased over the weeks so that after 2 months of HFD feeding, lacDrd2KO female mice had increased their body weight by 30% and control mice by 14% (**Figure 2B**).

This differential increase in body weight in response to a HFD during the last weeks, could not be directly explained by food intake, inasmuch food intake did not increase during the eight weeks of the experiment in either genotype (**Figure 2C**). LacDrd2KO females showed higher food intake than diet-matched *Drd2 ^loxP/loxP^* mice (interaction time x genotype x diet F_7,126 =_ 0.96, *P*=0.46, NS; main effect of genotype, *P*=0.0087, **Figure 2B**). Importantly, significantly more fat was consumed by HFD lacDrd2KO compared to HFD *Drd2^loxP/loxP^* females throughout the experiment (main effect genotype, *P*=0.0059, HFD lacDrd2KO vs. HFD *Drd2 ^loxP/loxP^*, **Figure 2D**). Caloric intake did not differ between diets for each genotype, but increased food intake in lacDrd2KO mice resulted in increased caloric intake in lacDrd2KO vs. *Drd2 ^loxP/loxP^* diet-matched mice (**Figure 2E**).

### Effect of Hyperprolactinemia on High-Fat Diet Impact on Tissue/Organ Weight

**Figure 3:**
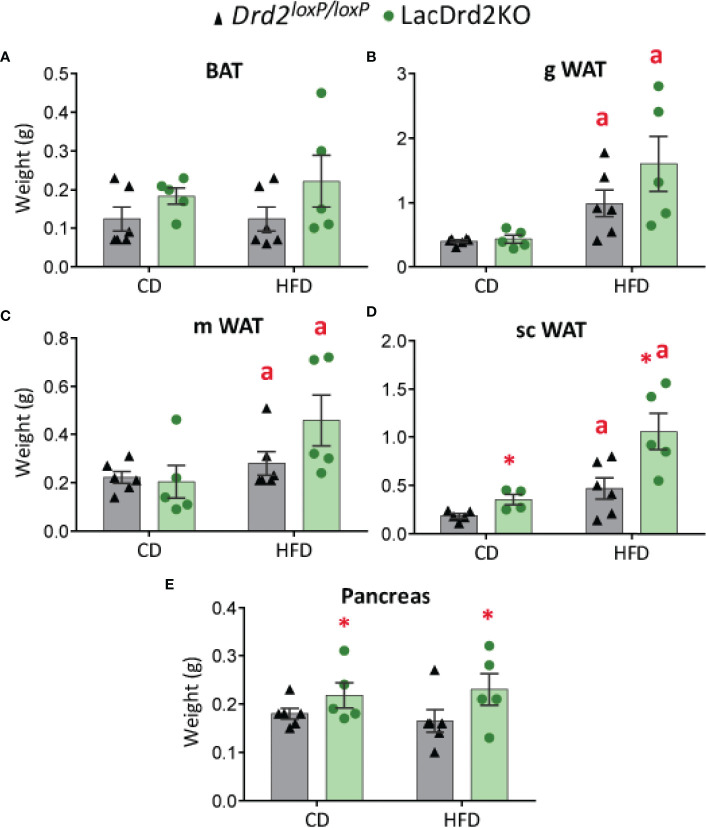
Effect of high-fat diet and hyperprolactinemia on tissue/organ weight. **(A)** brown adipose tissue (BAT), **(B)** gonadal white adipose tissue (gWAT), **(C)** mesenteric white adipose tissue (mWAT), and **(D)** subcutaneous white adipose tissue (scWAT) weights in g, **(E)** pancreas in 5-month-old *Drd2 ^loxP/loxP^* and lacDrd2KO female mice fed a CD or HFD for two months. N (for this figure the number of samples is defined from left to right within each panel) = 6, 5, 6, 5 **(A–C, E)**; and 5, 4, 6, 5 **(D)**. Two-way ANOVA; “a” = *P* ≤ 0. 05 vs. genotype- matched mice fed with control diet; *P ≤0.05 vs. diet- matched *Drd2 ^loxP/loxP^* mice.

Pancreas and WAT and BAT depots were dissected and weighed once the experimental protocol was completed, when animals were 5 months old. No significant differences were found between experimental groups in BAT depots at this age, though there was a tendency to increased BAT weight related to genotype but not to diet (main effect genotype *P*= 0.062, **Figure 3A**). Heavier visceral WAT depots were observed in HFD-fed animals regardless of genotype, while genotype did not modify these WAT depots (main effect diet: *P* = 0.0012, and *P* = 0.026 for gonadal and mesenteric WAT, respectively; and main effect of genotype *P* = 0.18 and 0.23 for gonadal and mesenteric WAT, respectively, **Figures 3B, C**). Interestingly, subcutaneous WAT depots were increased by diet and genotype (interaction genotype x diet, F_1,16 =_ 0.0019, *P*=0.60; main effect genotype, *P* = 0.0057, main effect of diet *P* = 0.0008; **Figure 3D**) suggesting an effect of high prolactin levels, as well as a potentiation of HFD-induced weight increase in this adipose tissue depot.

LacDrd2KO mice showed heavier pancreas compared to *Drd2 ^loxP/loxP^* mice for both diets (interaction genotype X diet, F_1,18 =_ 0.0019, *P*=0.97; main effect genotype, *P* = 0.043), while no diet-induced weight differences were observed for the pancreas (**Figure 3E**).

### Effect of Hyperprolactinemia on High-Fat Diet Induced Glucose Intolerance

**Figure 4:**
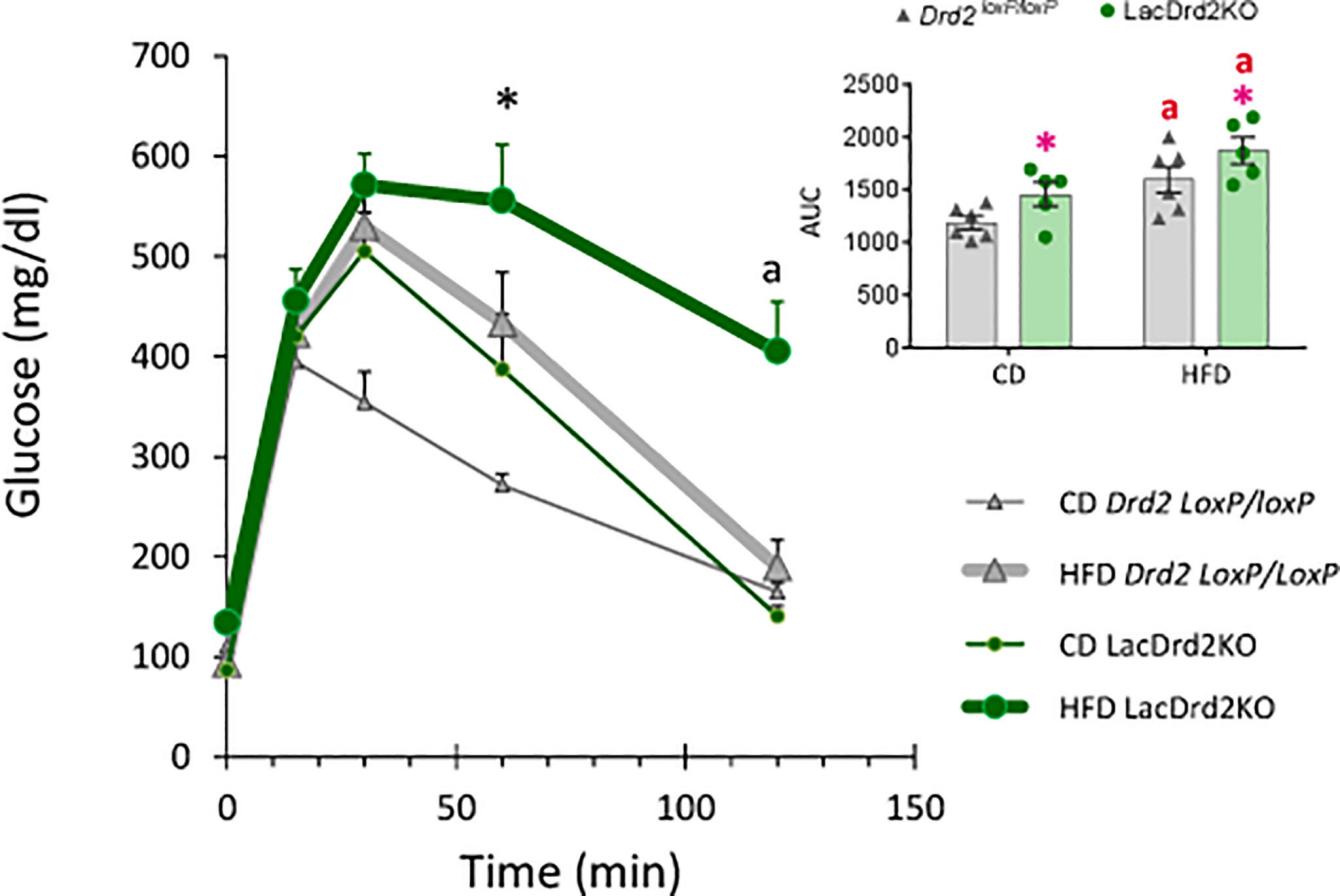
Effect of a high-fat diet and hyperprolactinemia on glucose homeostasis. Intraperitoneal glucose tolerance test (GTT, 2 mg/g) in fasted 5-month-old *Drd2 ^loxP/loxP^* and lacDrd2KO mice fed a CD or HFD. Three-way ANOVA with repeated-measures design for the effects of time, diet and genotype; “a” *P*< 0.01 vs. time 0 for genotype- and diet- matched mice, and *P <0.01 vs. time-matched *Drd2 ^loxP/loxP^* CD mice, n = 6,6, for *Drd2 ^loxP/loxP^* mice fed control diet and HFD, respectively, and n=5,5 for lacDrd2KO mice fed a control diet and a HFD. Inset: Area Under the Curve (AUC). Two-way ANOVA; “a” = *P* ≤ 0. 05 vs. genotype- matched mice fed with control diet; *P ≤0.05 vs. diet- matched *Drd2 ^loxP/loxP^* mice.

Glucose metabolism was next investigated *in vivo* two weeks before the end of the protocol. A glucose tolerance test (GTT) indicated that LacDrd2KO mice on a control diet were glucose intolerant compared to *Drd2 ^loxP/loxP^ mice* (**Figure 4**). Furthermore, exposure to a HFD for 8 weeks also induced glucose intolerance in *Drd2 ^loxP/loxP^* mice. Interestingly, we found that hyperprolactinemia deepened the alterations in glucose metabolism observed in mice exposed to a HFD, because after 120 minutes, blood glucose levels remained higher compared to genotype- and diet-matched mice at time zero only in the lacDrd2KO-HFD group (Three Way ANOVA, interaction time X genotype X diet F_4,72 =_ 2.94, *P*=0.026; *P* time 120 vs. time 0 = 0.98, 0.14, 0.99 and 0.00019 for CD-*Drd2 ^loxP/loxP^*, HFD-*Drd2 ^loxP/loxP^*, LacDrd2KO-CD, and HFD-lacDrd2KO mice, respectively (**Figure 4**). Besides, at 60 min post injection glucose levels were significantly higher compared to CD-*Drd2 ^loxP/loxP^* only in HFD-lacDrd2KO mice (P= 0.0013, 0.73 and 0.10 for LacDrd2-HFD, LacDrd2-CD and HFD-*Drd2 ^loxP/loxP^* vs CD-*Drd2 ^loxP/loxP^* at 60 min post glucose injection*).* When we analyzed area under the curve (AUC) we found a genotype and a diet effect (interaction diet x genotype, F_1,18 =_ 0.001, *P*=0.97; main effect of diet *P* = 0.0012, main effect of genotype *P <*0.021; inset), and the differences in glucose clearance could not be detected when all time points were considered together.

### Impact of Hyperprolactinemia on Subcutaneous White Adipose Tissue Gene Expression Profile in lacDrd2KO and *Drd2 ^loxP/loxP^* Mice Fed Control or High-Fat Diet

**Figure 5:**
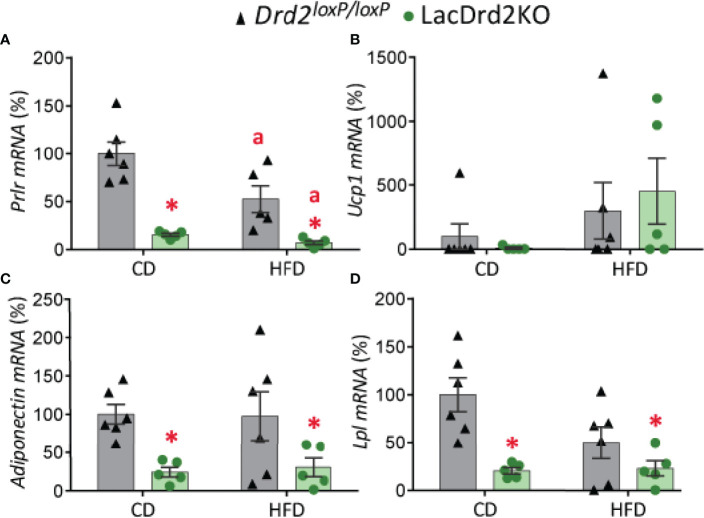
Impact of hyperprolactinemia and high-fat diet on gene expression profile of subcutaneous white adipose tissue. mRNA levels of **(A)** *Prlr*, **(B)** *Ucp1*, **(C)** adiponectin and **(D)** *Lpl*, in 5- month-old *Drd2 ^loxP/loxP^* and lacDrd2KO female mice with free access to a control diet (CD) or a HFD. *Drd2 ^loxP/loxP^* mice (CD, n = 5; HFD, n = 6), lacDrd2KO mice (CD, n = 5; HFD n = 5). Two-way ANOVA; “a” =*P* ≤ 0. 05 vs. genotype-matched mice fed with control diet, * *P* ≤0.05 vs. diet-matched *Drd2 ^loxP/loxP^* mice.

Exposure to a HFD and hyperprolactinemia decreased *Prlr* gene expression in scWAT (interaction diet x genotype, F_1,17 =_ 3.85, *P*=0.067; main effect of diet *P* = 0.012, main effect of genotype *P <*0.0001; **Figure 5A**).

Low basal expression levels of *Ucp1* were found in scWAT depots in both genotypes, and exposure to a HFD increased gene expression levels of this thermogenic marker only in some samples (*P* = 0.084; **Figure 5B**).

Expression levels of adiponectin and lipoprotein lipase (*Lpl*), two genes implicated in lipid metabolism, were significantly lower in lacDrd2KO female mice compared to their *Drd2 ^loxP/loxP^* control counterparts, regardless of the type of diet received (for adiponectin: interaction genotype x diet, F_1,18_ = 0.081, *P*=0.45; main effect genotype, *P* = 0.0022; for *Lpl*: interaction genotype x diet, F_1,18_ = 3.64, *P*=0.072; main effect genotype, *P* = 0.0012; **Figures 5C, D**).

### Impact of Hyperprolactinemia on Brown Adipose Tissue Gene Expression Profile in lacDrd2KO and *Drd2 ^loxP/loxP^* Mice Fed Control or High-Fat Diet

**Figure 6:**
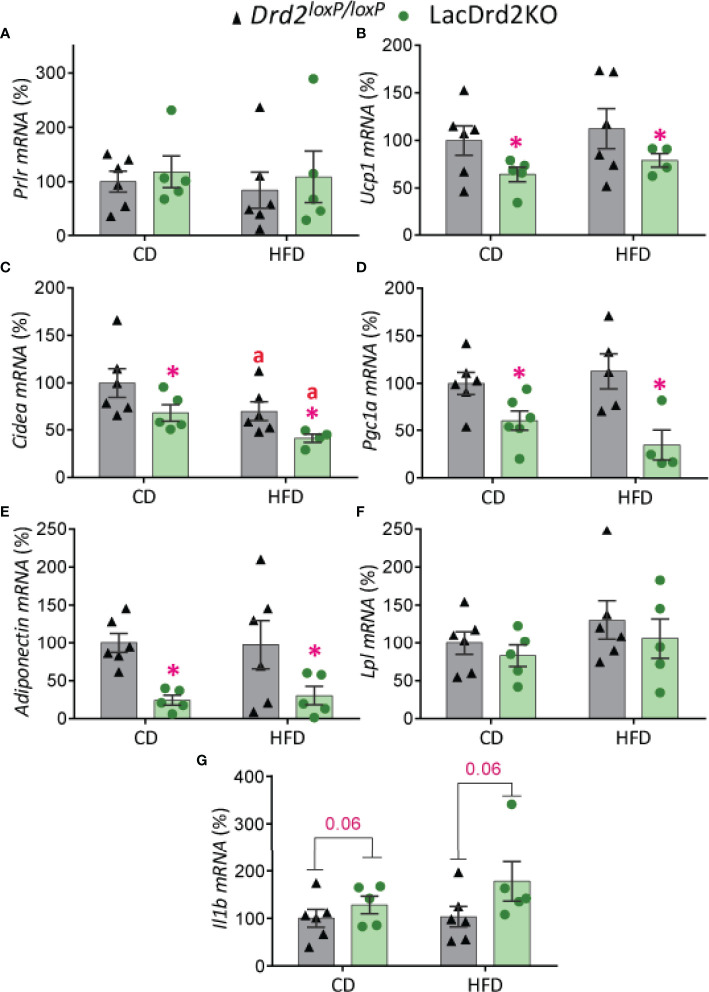
Impact of hyperprolactinemia and high fat diet on gene expression profile of brown adipose tissue. mRNA levels of **(A)** *Prlr*, **(B-D)** *Ucp1*, *Cidea* and *Pgc1a*, indicators of thermogenic capacity **(E)** adiponectin, **(F)** *Lpl*, and **(G)** *Il1b* in *Drd2 ^loxP/loxP^* and lacDrd2KO 5-month-old female mice with free access to a control diet (CD) or a HFD during two months. *Drd2 ^loxP/loxP^* (CD, n = 6; HFD, n = 6), lacDrd2KO (CD, n = 5; HFD, n = 5). Two-way ANOVA; “a” *P* ≤ 0. 05 vs. genotype-matched mice fed with control diet, *P ≤0.05 vs. diet-matched *Drd2 ^loxP/loxP^* mice.

There was a marked effect of hyperprolactinemia on mRNA levels of thermogenic- related genes in BAT, though no significant differences were found between experimental groups in the expression levels of *Prlr* (**Figure 6A**). Regardless of the diet received, LacDrd2KO female mice had significantly lower expression of *Ucp1* (interaction genotype x diet, F_1,17_ = 0.0035, *P*=0.95; main effect genotype, *P* = 0.045), *Cidea* (interaction genotype x diet, F_1,17_ = 0.011, *P*=0.92; main effect genotype, *P* = 0.018) and *Pgc1a* (interaction genotype X diet, F_1,17_ = 1.8, *P*=0.19; main effect genotype, *P* = 0.0007) compared to *Drd2^loxP/loxP^* control mice (**Figures 6B–D**). Furthermore, a diet effect on *Cidea* mRNA expression levels was detected for both genotypes (main effect of diet *P* = 0.024), and therefore the combination of HFD and high prolactin levels further decreased *Cidea* mRNA expression levels (**Figure 6C**).

The expression levels of genes involved in lipid metabolism indicated that exposure to a HFD did not alter adiponectin mRNA levels in BAT but hyperprolactinemia significantly decreased its expression levels in control and HFD fed mice (interaction genotype X diet, F_1,17 =_ 0.045, *P*=0.83; main effect genotype, *P* = 0.0022, **Figure 6E**). On the other hand, no significant differences between experimental groups were evidenced for *Lpl* expression levels in this adipose tissue depot (**Figure 6F**). Interestingly, hyperprolactinemia but not fat diet showed a strong tendency to increase the proinflammatory cytokine interleukin-1b *Il1b* (interaction genotype X diet, F_1,18 =_ 0.78, *P*=0.39; main effect genotype, *P* = 0.064, main effect of diet *P*=0.32, **Figure 6G**).

### Histological Analysis of Interscapular Brown Adipose Tissue in lacDrd2KO and *Drd2^loxP/loxP^* Mice Exposed to Control or High-Fat Diet

**Figure 7:**
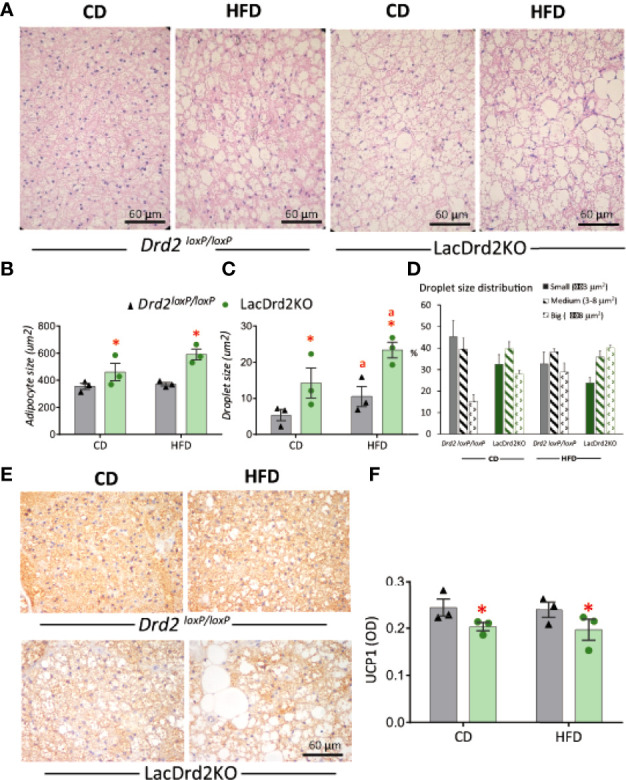
Impact of hyperprolactinemia and high fat diet on brown adipose tissue architecture, and UCP1 staining **(A)** H&E staining of paraffin embedded samples of BAT from Drd2 *^loxP/loxP^* and lacDrd2KO female mice fed with control diet (CD) or HFD. Representative images captured at 40X magnification using light microscopy. **(B)** Average adipocyte size (μm^2^) in BAT tissue from *Drd2 ^loxP/loxP^* and lacDrd2KO female mice fed with control diet (CD) or HFD, n=3 for each group, *P = 0.0036 vs. *Drd2 ^loxP/loxP^* diet-matched mice; **(C)** Average droplet size (μm^2^) within brown adipocytes from *Drd2 ^loxP/loxP^* and lacDrd2KO female mice fed with control diet (CD) or HFD, n = 3 for each group, *P = 0.0051 vs. *Drd2 ^loxP/loxP^* diet-matched mice; “a” *P* = 0.037 vs CD, genotype-matched mice; **(D)** Percentage of droplets according to size (small ≤ 3 μm^2^, medium from 3.01 to 8 μm^2^, and big ≥ 8.01 μm^2^ in the four experimental groups; **(E)** Representative images of UCP1 staining, **(F)** quantification of immunoreactive UCP1 from BAT samples from *Drd2 ^loxP/loxP^* and lacDrd2KO female mice fed with control diet (CD) or HFD; n = 3 for each group, * *P* = 0.041 vs. *Drd2 ^loxP/loxP^* diet-matched mice.

Hematoxylin and eosin staining revealed that hyperprolactinemia induced marked changes in BAT morphology (**Figure 7A**). Adipocyte size was increased in hyperprolactinemic mice compared to *Drd2 ^loxP/loxP^* pair-fed mice (interaction diet x genotype F_1,8 =_ 2.02, *P*=19, effect of genotype *P*=0.036, **Figure 7B**). The morphometric analysis assessing the size of lipid droplets within adipocytes pointed to a morphological transition to larger lipid droplets induced by hyperprolactinemia (interaction diet x genotype F_1,8_ = 0.49, *P*=0.50; main effect diet *P*= 0.037, main effect genotype *P*= 0.0051), and therefore a greater increment was evidenced when the two challenges were combined (**Figure 7C**), pointing to a process of whitening of the tissue evoked by hyperprolactinemia and HFD. The analysis of the proportion of small, medium and large lipid droplets demonstrated a marked reduction of small fat droplets and a concomitant increase in large droplets in both wildtype-HFD and LacDrd2KO-CD compared to wildtype-CD, indicating an independent effect of high prolactin levels on droplet size. When both challenges were combined there was a further increase in large and decrease in small droplets (**Figure 7D**).

In addition, immunohistochemical staining for UCP1 in BAT sections showed a clear correlation between transcript and protein expression levels. Lower protein levels of UCP1 were observed in hyperprolactinemic females irrespective of diet (interaction diet x genotype F_1,8 =_ 0.0016, *P*=0.97, main effect of genotype *P* = 0.041; **Figures 7E, F**). Overall, these results indicate a role for prolactin in the morphologic transition of BAT to a white adipose-like tissue with lower expression of thermogenic markers.

## Discussion

Different experimental settings indicate that hyperprolactinemia is associated with weight gain in part as a result of increased food intake. For example, in female rats, drug-induced hyperprolactinemia, or 10-day prolactin administration, as well as ectopic pituitary transplants, increase food intake and body weight (5–7). Furthermore, previous evidence from our laboratory showed that hyperprolactinemic lacDrd2KO are overweight, but, despite early occurrence of hyperprolactinemia, obesity was gradual and of late onset beginning mildly in 6-month-old females and evolving to frank obesity at 10 months of age (9). These data are in accordance with clinical data, which show that not all patients with prolactinomas are obese. Because obesity and food intake behaviors are multifactorial in origin, we sought to combine high prolactin levels and a HFD in order to establish whether metabolic changes could be accelerated or potentiated by combining the metabolic challenges, and furthermore we wished to dissect the role of prolactin on BAT function in this situation. We therefore exposed the life-long hyperprolactinemic mouse model to CD or HFD for two months, starting at 3 months of age, and characterized the metabolic role of prolactin on brown and white adipose tissue depots and glucose homeostasis, in a context of normal or increased energy availability.

Food intake is regulated by an integrated system involving both homeostatic and hedonic brain circuits sensitive to peripheral factors (42). Homeostatic regulation of food intake comprises hypothalamic systems that drive food intake when energy stores are depleted, whereas hedonic or reward-based regulation is driven by dopaminergic pathways that may override homeostatic satiety signals during periods of relative energy abundance by increasing the desire to consume highly palatable, energy-dense food, when extra calories are unnecessary (43, 44). Our results show that alterations in food intake induced by prolactin may be associated with altered homeostatic and hedonic brain circuits. In this respect, the role of prolactin in controlling hedonic feeding has not been explored to date.

We detected effects for both diet and high prolactin levels on body weight progression. HFD increased body weight in both lacDrd2KO and control female mice, but importantly, this increase was evidenced much earlier in the hyperprolactinemic group, and the difference in body weight between genotypes increased significantly over time, clearly suggesting that chronic high prolactin levels aggravate body weight gain induced by HFD.

Increased food intake and body weight gain affected adipose tissue accretion. The identification of adipose tissue as a metabolic organ has achieved increasing importance in recent years as its dysfunction is considered one of the central events that triggers the development of obesity. In a context of positive energy balance favored by HFDs, the storage capacity of scWAT is limited, and ectopic fat accumulation can occur in other tissues such as liver, skeletal muscle and heart, or in VAT depots (45). Excessive ectopic lipid accumulation in VAT depots predisposes to the development of metabolic syndrome, defined by a cluster of interconnected factors that directly increase the risk of coronary heart disease, other forms of cardiovascular atherosclerotic diseases, and diabetes mellitus type 2 (46, 47). The role of prolactin in fat accumulation in the different adipose tissue depots has not been extensively studied, and our results dissect a differential impact of prolactin for each fat depot.

In accordance with other models of diet-induced obesity (48), mice of both genotypes exposed to a HFD for two months had significantly larger scWAT and visceral fat pads, the latter including gonadal and mesenteric adipose tissue. Interestingly, lacDrd2KO mice fed a control diet had heavier scWAT depots compared to their control counterparts, and exposure to HFD potentiated scWAT weight gain in this group, suggesting an impact of elevated prolactin levels specifically on scWAT weight.

To this respect, it has been described that scWAT is a metabolically plastic tissue, sensitive to the action of prolactin (49). *Prlr* knockout mice have impaired development of scWAT depots due to fewer adipocytes, indicating a role for prolactin on adipogenesis in this tissue (28). In this context, significant changes in the expression levels of genes related to metabolism were detected in scWAT of lacDrd2KO mice. *Prlr* mRNA levels were significantly reduced independently by genotype and diet. These results suggest that chronic hyperprolactinemia can lead to adipose tissue dysfunction and limit the effect of prolactin in this tissue; in contrast, prolactin induces its own receptor in most tissues (25). Results are consistent with previous evidence obtained from microarray studies in adipose tissue that demonstrated that *Prlr* is downregulated in diet-induced obese mice (50). Interestingly, HFD increased the expression levels of *Ucp1* in scWAT in some samples in both genotypes, probably indicating heterogeneity of samples with regard to a beiging process. Previous evidence showed that a hypercaloric diet may induce the appearance of thermogenic adipocytes in WAT depots, particularly in scWAT, contributing to counteract the excess calories incorporated with the fat-enriched diet (51), but on the contrary, HFD may induce the whitening of beige depots (52). It has been described that the absence of prolactin stimulation in *Prl* knockout mice induces the appearance of beige cells with thermogenic capacity in perirenal adipose depots in response to HFD (49), but we did not observe changes in *Ucp1* mRNA expression in scWAT depots related to genotype. Nevertheless, prolactin may impact on UCP1 independent mechanisms which contribute to the thermogenic action of white adipocytes, such as creatine-substrate cycling and Ca2+cycling (53). On the other hand, we found a strong inhibitory effect of prolactin and not HFD on *Lpl* and adiponectin expression levels in scWAT, which may suggest an additional mechanism participating specifically in prolactin-induced obesity. In concordance, it has been shown that elevated serum prolactin levels decrease mRNA expression of *Lpl* and adiponectin in adipocytes, *in vivo* and *in vitro*, and may contribute to the development of an obese phenotype (26, 54). LPL is the rate-limiting enzyme for the uptake and storage of triglyceride-derived fatty acids by adipose tissue (55). Reduced *Lpl* expression levels found in lacDrd2KO female mice regardless of the diet received, suggest that during chronic hyperprolactinemia scWAT depots may compensate adipocyte hypertrophy by limiting lipid uptake and storage capacity (56). On the other hand, adiponectin, a hormone synthesized and released into circulation by adipose tissue, regulates energy homeostasis playing an important role in the dialogue between adipose tissue and other metabolic tissues or organs (57). Decreased adiponectin levels in scWAT induced by high prolactin levels and not HFD, may be relevant to obesity and intolerance to glucose evidenced in lacDrd2KO mice, supporting the described role for low adiponectin levels in tissue resistance to insulin, and/or increased adiposity in obese patients, pigs and rodents (57–59).

Our results uncover a role for prolactin in BAT thermogenic gene expression, as well as an interaction with the effect of HFD in this adipose tissue. Plenty of evidence shows that obesity is associated with an increase in BAT weight, explained by a higher lipid content and a reduction in its thermogenic function (33, 60, 61). In this sense, heavier BAT depots do not necessarily indicate a greater thermogenic capacity but, instead, are associated with a defective tissue, as triglycerides are stored instead of being used for heat production (33).

Histological analysis of this tissue revealed increased size of lipid droplets within brown adipocytes, in response to either hyperprolactinemia or HFD independently, and, importantly, prolactin further increased lipid droplet size after exposure to an HFD. To be noted is that increased brown adipocyte size was evidenced in hyperprolactinemic non-obese CD fed mice. Therefore, the histomorphological alterations observed in BAT depots in the presence of prolactin suggest that the marginal BAT weight gain observed in lacDrd2KO mice could be associated to a lipid accumulation probably associated to a decrease in its thermogenic capacity (33), and that prolactin plays a major role.

During lactation, when prolactin levels are high, the expression levels of thermogenic genes in BAT decrease (62), indicating a negative association between prolactin and the thermogenic capacity of this tissue. Moreover, *Prlr* knockout mice fed a HFD for 16 weeks show resistance to weight gain compared to control mice partly due to increased expression levels of thermogenic markers in BAT (*Ucp1, Pgc1a*) and beiging in perirenal VAT (49), indicating improved thermogenesis in the absence of prolactin action. A negative association between high prolactin and the thermogenic capacity of BAT has also been proposed as a physiological adaptation during pregnancy and lactation, when glucose and triglycerides should not be burned to produce heat because they are needed for the pregnant and lactating mother, and glucose for the developing fetus. Furthermore, lactation performance is influenced by the capacity to dissipate body heat, and prolactin may participate in this process limiting thermogenesis (62).

Immunohistochemical and gene expression analysis of thermogenic markers of BAT, confirmed our histological observations that pointed to an alteration of the tissue in response to high prolactin levels. We studied UCP1 as well as cell death-inducing DFFA-like effector A (*Cidea*) and peroxisome proliferator activated receptor γ coactivator 1α (*Pgc1a*), two genes abundantly expressed in brown and beige adipocytes, which are critical regulators of BAT activity in response to environmental stimuli such as cold temperature and diet (63, 64). Significant decrements in the expression of *Ucp1* and *Pgc1a* mRNA as well as UCP1 protein levels induced by prolactin but not by HFD, indicate that prolactin, independently of the diet or obesity, may predispose to the development of an obese phenotype by lowering the thermogenic capacity of BAT. According to previous evidence, expression levels of *Ucp1* and *Pgc1a* are positively correlated because the activation of Pgc1*α* pathway induces mitochondrial biogenesis consequently increasing *Ucp1* gene expression in BAT. *Cidea* was decreased by both prolactin and HFD, and the lowering impact of prolactin on BAT *Cidea* expression was significantly intensified when lacDrd2KO mice were exposed to a HFD, indicating a synergy of effects.

The observed reduction in the levels of expression of the typical markers of BAT, exclusively in hyperprolactinemic non obese and not in HFD fed mice point to an action of prolactin independently of food intake driven-obesity. These changes are concordant with quantitative analysis of histological sections which showcase the occurrence of BAT whitening, associated with an accumulation of large lipid droplets due to mitochondrial dysfunction and loss (33), and decreased expression levels of *Ucp1*, *Pgc1a* and *Cidea*. Collectively, these data suggest a negative action of prolactin on BAT thermogenic capacity, and point to a new avenue of prolactin action.

During BAT whitening there is a proneness to tissue inflammation and ER stress (34–36), increased autophagy (65, 66), and insulin resistance of the tissue (67, 68). Prolactin has been associated with inflammation in other tissues (69) but there are no data regarding an action of prolactin on BAT inflammation, ER stress or autophagy. In the present work, increased tendency in BAT mRNA levels of the proinflammatory cytokine *Il1b* suggest that prolactin may evoke inflammation of the tissue. Furthermore, a negative correlation of BAT UCP1 levels and NPY expression in the dorso medial hypothalamus has been described (70), and we previously showed that high prolactin levels increase *Npy* expression in the DMH (38) probably favoring food intake, and simultaneously decreasing BAT *Ucp1*, pointing to an indirect mechanism of action or prolactin on BAT function. Finally, the decrease in adiponectin expression levels in BAT in response to hyperprolactinemia and not HFD suggests a lower contribution to adiponectin plasma levels that could partly participate in the greater weight gain, given that significant negative associations have been described between adiponectin levels and body mass index (59, 71). Alternatively, obesity could be the cause of the low adiponectin expression levels.

An increase in pancreas weight was evidenced in lacDrd2KO female mice regardless of the type of diet received, suggesting a strong impact of elevated serum prolactin levels in the physiology of this organ. We have previously demonstrated that lacDrd2KO female mice fed a regular diet are hyperinsulinemic, and have increased pancreatic insulin content (27). This may be related to the increased tissue weight observed in the present experiments, and is in line with the role described for prolactin in pancreas development and proliferation (3). Glucose intolerance was observed in response to HFD or chronic hyperprolactinemia separately, and importantly, intolerance was exacerbated in HFD animals that were also hyperprolactinemic, suggesting an aggravating effect of prolactin on altered glucose homeostasis induced by HFD. To this respect, it has been described that prolactin administered in high doses alters the signaling mechanism through which glucose enters pancreatic β cells and therefore disrupts adequate insulin response to glucose which may finally lead to glucose intolerance (72) (27).

In conclusion, chronic high prolactin levels accelerate and aggravate the metabolic effects of exposure to a HFD, and have an independent impact on BAT. In normal weight hyperprolactinemic mice, exposure to a HFD enhanced body weight gain, glucose intolerance, and scWAT weight gain, to a greater degree than in control mice. In particular, the combination of metabolic insults (elevated prolactin and HFD) altered BAT induced marked tissue whitening and a pronounced decrease in the levels of expression of markers of thermogenic capacity. Furthermore, there was a synergy in the decrease of expression levels of *Cidea* in BAT in response to the combination of metabolic challenges, which further points to a mechanism involved in the worsening of the obese phenotype. But interestingly many metabolic alterations were mainly associated to high prolactin levels, and not to HFD or increased BW in our protocol, such as the decrease in mRNA levels of adiponectin and *Lpl* in scWAT, and of *Ucp1*, *Pgc1a* and adiponectin in BAT, pointing to an independent effect of prolactin.

Since the description of active and recruitable BAT in humans, increasing interest in its physiology and pathology has positioned this tissue in a new hierarchy as a possible target to tackle metabolic dysfunction. In this context, deciphering prolactin action, and the effect of prolactin antagonism on brown adipocytes may yield valuable tools in the effort to curtail obesity or accelerate weight loss.

Our results demonstrate that metabolic worsening can result from the combination of metabolic challenges. Therefore, even though prolactin is fundamental in orchestrating adaptive changes in metabolism during pregnancy and lactation, and physiological or slightly high levels of prolactin have a protective metabolic role, its pathological increase could be detrimental in an obesogenic environment that requires greater energy expenditure and not increased calorie intake. In particular, prolactin targets BAT inducing a decreased thermogenic capacity. These results should be considered in case of prolactinomas, or chronic psychiatric treatments that target the DRD2 and evoke chronic hyperprolactinemia. In this context, prolactin may represent an additional player in the development of obesity, which is multifactorial in origin, and therefore the interrelation of genetic, endocrine, behavioral and environmental factors deserves greater attention in the search of strategies to combat overweight.

## Data Availability Statement

The raw data supporting the conclusions of this article will be made available by the authors, without undue reservation.

## Ethics Statement

The animal study was reviewed and approved by Institutional animal care and use committee of the Institute of Biology and Experimental Medicine, Buenos Aires in accordance with the Animal Welfare Assurance for the Institute of Biology and Experimental Medicine, Office of Laboratory Animal Welfare, NIH, A#5072-01.

## Author Contributions

FL-V Conceived and designed research, performed experiments, analyzed data, interpreted results of experiments, prepared figures, drafted manuscript, edited and revised manuscript, approved final version of manuscript. CW performed experiments, analyzed data, interpreted results of experiments, edited and revised manuscript, approved final version of manuscript. ES Conceived and designed some experiments, analyzed data, edited and revised manuscript, approved final version of manuscript. AO performed experiments *in vivo*, approved final version of manuscript. JT performed immunohistochemical studies, approved final version of manuscript. DB-V Conceived and designed research, analyzed data, interpreted results of experiments, prepared figures, drafted manuscript, edited and revised manuscript, approved final version of manuscript. All authors contributed to the article and approved the submitted version.

## Funding

This work was provided with financial support for the conduct of the research by Argentinean Agency for Promotion of Science and Technology (PICT 2016-526 and 2019-1619 to DB-V), National Research Council (CONICET, PIP 2021 #273) and Williams and Rene Baron Foundations to DB-V. FL-V and CW received a fellowship from the CONICET.

## Conflict of Interest

The authors declare that the research was conducted in the absence of any commercial or financial relationships that could be construed as a potential conflict of interest.

## Publisher’s Note

All claims expressed in this article are solely those of the authors and do not necessarily represent those of their affiliated organizations, or those of the publisher, the editors and the reviewers. Any product that may be evaluated in this article, or claim that may be made by its manufacturer, is not guaranteed or endorsed by the publisher.
